# Crystal Structure of the Formin mDia1 in Autoinhibited Conformation

**DOI:** 10.1371/journal.pone.0012896

**Published:** 2010-09-30

**Authors:** Takanori Otomo, Diana R. Tomchick, Chinatsu Otomo, Mischa Machius, Michael K. Rosen

**Affiliations:** 1 Department of Biochemistry, University of Texas Southwestern Medical Center at Dallas, Dallas, Texas, United States of America; 2 Howard Hughes Medical Institute, University of Texas Southwestern Medical Center at Dallas, Dallas, Texas, United States of America; University of Oulu, Germany

## Abstract

**Background:**

Formin proteins utilize a conserved formin homology 2 (FH2) domain to nucleate new actin filaments. In mammalian diaphanous-related formins (DRFs) the FH2 domain is inhibited through an unknown mechanism by intramolecular binding of the diaphanous autoinhibitory domain (DAD) and the diaphanous inhibitory domain (DID).

**Methodology/Principal Findings:**

Here we report the crystal structure of a complex between DID and FH2-DAD fragments of the mammalian DRF, mDia1 (mammalian diaphanous 1 also called Drf1 or p140mDia). The structure shows a tetrameric configuration (4 FH2 + 4 DID) in which the actin-binding sites on the FH2 domain are sterically occluded. However biochemical data suggest the full-length mDia1 is a dimer in solution (2 FH2 + 2 DID). Based on the crystal structure, we have generated possible dimer models and found that architectures of all of these models are incompatible with binding to actin filament but not to actin monomer. Furthermore, we show that the minimal functional monomeric unit in the FH2 domain, termed the bridge element, can be inhibited by isolated monomeric DID. NMR data on the bridge-DID system revealed that at least one of the two actin-binding sites on the bridge element is accessible to actin monomer in the inhibited state.

**Conclusions/Significance:**

Our findings suggest that autoinhibition in the native DRF dimer involves steric hindrance with the actin filament. Although the structure of a full-length DRF would be required for clarification of the presented models, our work here provides the first structural insights into the mechanism of the DRF autoinhibition.

## Introduction

Formins are conserved actin regulators, which function in formation of stress fibers, actin cables, the cytokinetic ring and filopodia [1]–[4]. Unlike the branched network of short actin filaments generated by the Arp2/3 complex [5]–[8], formin activity results in unbranched, long filaments in cells [9], [10]. Formins modulate actin dynamics through two biochemical activities. They accelerate nucleation of new filaments *de novo* from actin monomers. Following nucleation they also remain stably associated at the barbed-ends of growing filaments as actin monomers are added, in a process termed “processive capping” [10]–[15]. Both in cells and *in vitro*, processive capping allows formins to protect the filament barbed ends from capping proteins and to modulate rates of filament elongation [11], [12], [16], [17].

**Figure 1:**
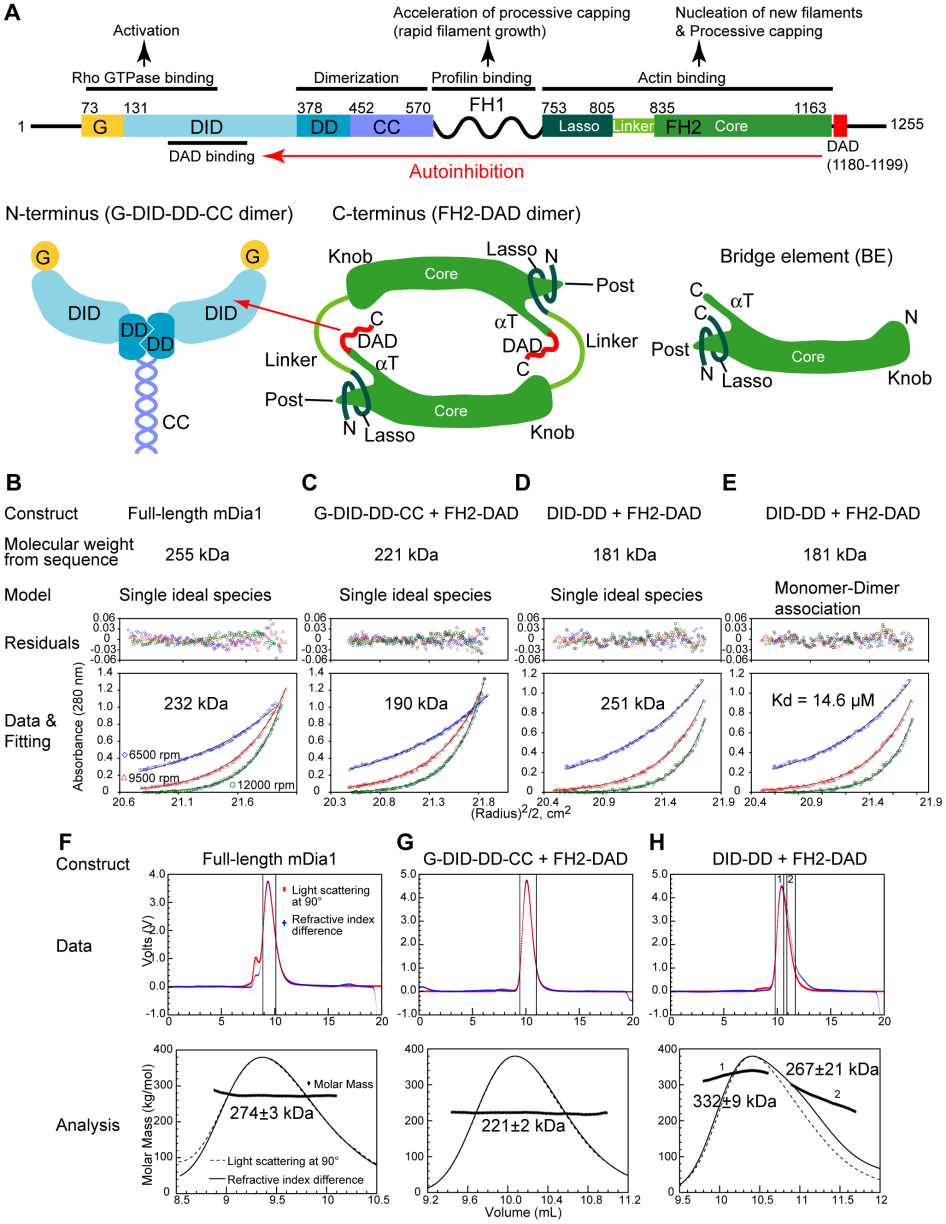
Domain organization and oligomerization of mDia1. (A) Domain organization of mDia1. Abbreviations: G, GTPase binding region necessary for RhoA binding; DID, Diaphanous inhibitory domain; DD, dimerization domain; CC, coiled coil; FH1, formin homology 1 domain; FH2, formin homology 2 domain; DAD, Diaphanous autoinhibitory domain. (B-E) Sedimentation equilibrium analyses of (B) full-length protein, (C) the G-DID-DD-CC•FH2-DAD and (D, E) the DID-DD•FH2-DAD complex. Representative data recorded for each protein/complex with a sample concentration of 14 µM at 6500 (blue diamonds), 9500 (red triangles), and 12000 rpm (green circles) are shown. The curves were fitted with single ideal species or monomer model in (B-D) and monomer-dimer equilibrium model in (E), respectively. In (E), the molecular weight of the monomer was fixed to 181kDa, which corresponds to the sum of two chains of each of DID-DD and FH2-DAD. The dissociation constant obtained from the fitting is 14.6 µM. Above these data are the residuals between the data and the fitted lines. (F-H) Size exclusion chromatography (Superdex 200) coupled multi-angle static light scattering experiments for (F) full-length protein, (G) the G-DID-DD-CC•FH2-DAD and (H) the DID-DD•FH2-DAD complex. Representative signals from the 90° detector (red) and refractive index differences (blue) are shown (upper panels). The two vertical solid lines indicate the regions used for calculating average molar mass (upper panels). The calculated molecular weights for each data point are shown as black diamonds in the expanded pictures of the selected regions (lower panels). 500 µl of total 25 µM protein were injected.

These actin regulatory activities are carried out by the conserved formin homology 2 (FH2) domain [18], [19]. Crystal structures of the FH2 domain of the yeast formin Bni1p in isolation and in complex with actin have shed light on structural mechanisms of FH2 function [20], [21]. The FH2 domain forms a ring-like dimer in which two structural units, referred to as actin bridge elements (BEs) or hemi-dimers, are connected through two flexible linkers (Fig. 1A). In the actin complex structure, each bridge element holds two actin monomers whose relative orientation is similar to the short-pitch dimer of an actin filament, suggesting nucleation through a templating mechanism [21]. Several different models for processive capping have been proposed, involving various modes of formin and actin fluctuations at the filament barbed end, which would allow the formin to remain persistently bound, while new monomers are added [11], [13], [21]–[25].

Diaphanous-related formins (DRFs) including mDia, Daam and FRL are effectors of Rho family GTPases, which function in processes such as cell migration, cytokinesis, cell polarity signaling, phagocytosis, and cancer metastasis [19], [26]–[30]. In isolation, DRFs are autoinhibited through binding of a C-terminal diaphanous autoinhibitory domain (DAD), which immediately follows the FH2 domain in sequence, and an N-terminal diaphanous inhibitory domain (DID) [31]–[33] (Fig. 1A). Autoinhibition is relieved by GTP-bound Rho proteins, which bind to the DID and an adjacent GTPase binding (G) element, and consequently displace the DAD. Structural studies have shown that the Rho and DAD binding sites on the DID are partially overlapped, explaining incompatibility of the two interactions [34]–[36]. The G-DID element of mDia1 is rendered dimeric through an adjacent dimerization domain (DD) and coiled coil region (CC) (Fig. 1A) [32], [34], [35]. Although the mechanisms of Rho-mediated activation of DRFs are reasonably clear, the mechanism of autoinhibition is not, in part because the FH2 domain shows no detectable binding to any other domains in DRF in isolation [32], [36]. This contrasts to other autoinhibited systems (e.g. WASP or Pak), in which activity bearing domains often bind to inhibitory domains directly with functionally critical residues being masked by the interaction [37]–[39].

Here we report the crystal structure of an autoinhibitory complex of mDia1 formed *in trans* between the DID-DD and FH2-DAD fragments of the protein. The structure exhibits a tetrameric configuration that contains four chains each of the DID-DD and FH2-DAD. Sedimentation equilibrium ultracentrifugation and multi-angle static light scattering (MALS) data indicate that the complex used for crystallization equilibrates between a dimer and a tetramer in solution. In contrast, full-length mDia1 and another complex formed *in trans* between FH2-DAD and a longer N-terminal fragment containing the DID-DD and an adjacent coiled-coil region are both dimers. The dimeric configurations that can be extracted from our crystal structure appear to be incompatible with filament binding, suggesting a likely mechanism of autoinhibition. However, additional NMR and biochemical analyses of a minimal element of this system (a monomeric bridge-DAD construct bound to DID) suggest that the autoinhibited structure may allow actin monomers to bind at least one of the two actin-binding sites on the FH2 domain.

## Results and Discussion

### Full-length mDia1 Forms a Dimer in Solution

Structural and biochemical studies have revealed that FH2 domains and the N-terminal regulatory fragment of mDia1 (G-DID-DD-CC, Fig. 1A) form dimers in isolation [32], [34], [35]. To determine if full-length mDia1 is also a dimer, we analyzed the intact protein as well as two intermolecular complexes of N- and C-terminal fragments (Fig. 1A) using sedimentation equilibrium ultracentrifugation and MALS. For full-length mDia1, the sedimentation equilibrium data can be described well by a single ideal species model, yielding a molecular mass of 232 kDa, which is reasonably close to the dimer mass (255 kDa) (Fig. 1B). The MALS-derived mass, 274 kDa, is also consistent with a predominantly dimeric species (Fig. 1F). For the intermolecular N+C complex of G-DID-DD-CC and FH2-DAD, both ultracentrifugation and MALS data give a mass consistent with two chains each of G-DID-DD-CC and FH2-DAD (190 kDa and 221 kDa, respectively, versus 221 kDa calculated mass) (Fig. 1C and G). Thus, full-length mDia1 and the complex of G-DID-DD-CC plus FH2-DAD produce analogous stoichiometries. In contrast, when the ultracentrifugation data on the complex of FH2-DAD plus DID-DD, a minimal dimeric inhibitory fragment, are fit to a single species model, the resulting mass (251 kDa) does not agree well with the calculated mass of the dimer (181 kDa) (Fig. 1D). However, the data fit well to a two species model ((DID-DD•FH2-DAD)_2_ + (DID-DD•FH2-DAD)_4_) with a dissociation constant of 14.6 µM (Fig. 1E). MALS data are also consistent with multiple species as indicated by a broad peak eluting from the prior gel filtration chromatography column, with masses ranging from 267 kDa (peak2) to 332 kDa (peak1) (Fig. 1H). The mass of 332 kDa is close to that of the (DID-DD/FH2-DAD)_4_ tetramer, 362 kDa. On this basis we conclude that the DID-DD•FH2-DAD complex likely equilibrates between a dimer and a tetramer in solution. Thus, the CC region appears to stabilize the dimer relative to the tetramer. Notably, the absence of the CC region does not affect the oligomerization state of the DID-DD fragment (Fig. S1) and G-DID-DD-CC and DID-DD inhibit FH2-DAD with equal potency in *in vitro* actin assembly assays (Fig. S2).

### Crystallization of an Autoinhibited Complex of mDia1

We attempted crystallization of mDia1 in the autoinhibited conformation using two separate chains of the N- and C-terminal domains, since the FH1 poly-proline region (∼200 residues) in the central segment of full-length mDia1 would likely interfere with crystallization. The FH2-DAD construct spanning residues 753–1209 was chosen based on previous structures of the FH2 and DAD region of various formins [36], [40], [41]. This was paired with a number of N-terminal constructs containing different lengths of the CC region. Only the construct completely lacking the CC region (residues 131–457, DID-DD) yielded crystals in complex with FH2-DAD. These diffracted to 2.75 Å under synchrotron illumination (Table 1). Phase information was obtained from a combination of a molecular replacement experiment and a multi-wavelength anomalous dispersion experiment collected on a selenomethionine-labeled sample.

**Table 1:** Data collection, phasing and refinement statistics for mDia1 DID-DD•FH2-DAD complex.

**Data collection**					
Crystal	SeMet #1 (Se^a^ *peak*)		SeMet #1 (Se^a^ *inflection point*)	SeMet #2 (Se^a^ *peak*)	Native
Wavelength (Å)	0.97937		0.97954	0.97946	0.97937
Resolution range (Å)	48.4–2.95 (3.00–2.95)		48.7–3.20 (3.27–3.20)	48.6 – 3.45 (3.5–3.45)	48.5–2.75 (2.81–2.75)
Unique reflections	102,553 (4,576)		81,256 (4,919)	65,944 (3,297)	123,292 (5,203)
Multiplicity	3.4 (2.3)		4.0 (2.6)	4.2 (4.2)	3.7 (2.3)
Data completeness (%)	97.1 (83.7)		99.2 (90.1)	100.0 (100.0)	95.4 (60.5)
*R*_merge_ (%)^b^	8.2 (39.3)		7.3 (62.9)	12.7 (56.1)	5.4 (50.1)
I/σ(I)	16.6 (1.6)		20.7 (1.5)	14.3 (2.7)	21.3 (1.4)
Wilson B-value (Å^2^)	89.5		103.2	89.7	84.0
**Phase determination**					
Anomalous scatterers		selenium, 15 out of 15 possible sites			
Figure of merit (35.0–2.95 Å)		0.20			
**Refinement statistics**					
Resolution range (Å)		29.9–2.75 (2.85–2.75)			
No. of reflections *R*_work_/R_free_		122,258/1,799 (8,435/124)			
Data completeness (%)		95.8 (67.0)			
Atoms (non-H protein)		24,224			
*R*_work_ (%)		19.9 (35.2)			
*R*_free_ (%)		26.1 (42.6)			
R.m.s.d. bond length (Å)		0.009			
R.m.s.d. bond angle (°)		1.35			
Mean B-value (Å^2^)		101.2			
Ramachandran plot (%) (favored/additional/disallowed)^c^		94.1/5.5/0.4			
Maximum likelihood coordinate error		0.45			
Missing residues, by chain		A: 453–457, 753, 806–828, 1196–1209.B: 131, 193–199, 753, 806–829, 1197–1209.C: 453–457, 753, 806–829, 1197–1209.D: 753, 806–829, 1197-1209.			

Data for the outermost shell are given in parentheses.

a Bijvoet-pairs were kept separate for data processing.

b *R*_merge_ = 100 Σ_h_Σ_i_|*I_h, i_*— 〈*I_h_*〉*|/*Σ*_h_*Σ_i_ *I_h,__i_*, where the outer sum (h) is over the unique reflections and the inner sum (i) is over the set of independent observations of each unique reflection.

c As defined by the validation suite MolProbity [55].

### Structure of an Autoinhibitory Complex of mDia1

**Figure 2:**
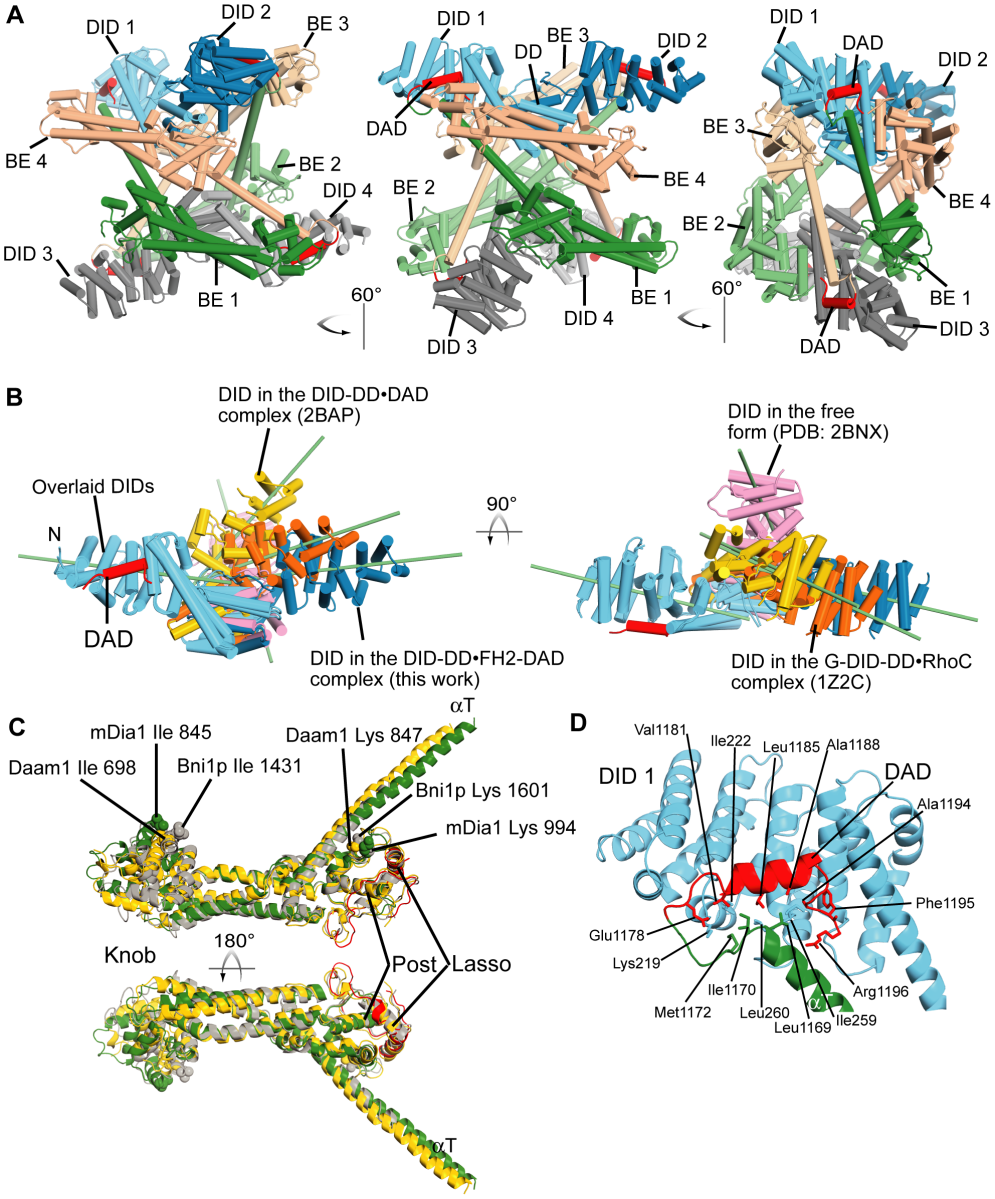
Crystal structure of autoinhibited tetrameric mDia1. (A) Structure of tetrameric mDia1 in three orientations. Abbreviation: BE, bridge element. (B) Overlay of the N-terminal inhibitory domain (DID-DD) of mDia1 from various crystal structures: the DID-DD•FH2-DAD complex (blue, this work), the free DID-DD-CC (pink, PDB code:2BNX), the DID-DD•DAD complex (yellow, 2BAP), and the RhoC-G-DID-DD complex (orange, 1Z2C). One of the two DIDs in each dimeric DID-DD structure is superimposed (left, sky blue DID) to illustrate the differences in position of the other DIDs. For clarity, the structures solved as complexes with RhoC and DAD, are shown without these binding proteins. The long axis of each DID molecule is indicated as a green bar. (C) An overlay of the Cα models of the bridge elements of mDia1 (green for the core and red for the lasso regions, this work), Daam1 (yellow, PDB code: 2J1D) and Bni1p (gray, 1Y64) is shown. The key residues that are critical for the activities and are identified by earlier works on Bni1p yeast formin (Ile 1431 in the Knob site and Lys 1601 in the Post site) [20], [21], [41] are shown in sphere model with their side-chains. The lysines in the Post site are well superposed but isoleucines in the Knob site are not. (D) An expanded view of the αT-DAD binding to DID. The orientation of the figure is similar to the middle of (A). The dotted line indicates the hypothetical connecting linker (5 residues 1172-1176) whose electron density is missing. The Ile 1170 contacts the hydrophobic residues of DID and DAD so that the peak corresponding this residue in the NMR experiment shows a shift from the free to the complex form (see also Fig. 4).

In the crystal (Fig. 2A), an asymmetric unit contains four chains each of DID-DD and FH2-DAD, consistent with the heavier mass observed in our ultracentrifugation and MALS data above. These are organized into two dimers of DID-DD (blue and grey in Fig. 2A), each of which is bound to a DAD element (red), and four bridge elements (dark/light green and dark/light tan). The linkers between bridge elements are not observed in the electron density, nor are the connections from bridge to DAD.

Within each (DID-DD)_2_ dimer, the structures of the individual sub-domains, and the contacts between the DDs are essentially the same as observed in previous structures of mDia1 N-terminal fragments (Fig. 2A and 2B) [34], [35], [40]. The DID is folded into a superhelical architecture with five repeats of a three helix element. The DD consists of three helices from each chain, which interdigitate with one another to create a dimer of six helices. The DID is bent slightly along its long axis. The pair-wise root mean square distances (r.m.s.d.s) of backbone heavy atoms between our structure and known mDia1 crystal structures are less than 1.5 Å for the DID and 2.2 Å for the DD in isolation (Table 2). However, the relative orientation between DID and DD is quite different among the available structures, resulting in differences in the relative positions of two DIDs of each (DID-DD)_2_ dimer (Fig. 2B). In the autoinhibited structure presented here, the long axes of the DID elements are roughly collinear, with a relative angle of 167°, and the DD dimer is slightly displaced perpendicularly to this axis. The two DID axes are related by 154° in the RhoC complex (PDB code:1Z2C), by 126° in the DAD complex (2BAP), and by 64° in the free structure (2BNX). The different orientations between DID and DD among the various crystal structures suggest that these elements likely sample a wide range of conformations in solution. Interestingly, of all the mDia1 N-terminal fragments crystallized to date, only the free DID-DD-CC exhibits an asymmetric conformation in the crystal [34]. This lack of symmetry may be important in producing dimers rather than tetramers in full-length mDia1 (Fig. 1).

**Table 2:** R.m.s.d. of backbone atoms of DID, DD, and DID-DD between the molecule A of the DID-DD•FH2-DAD structure in this work (PDB code: 3OBV) and available structures.

PDB code	DID (135–377)	DD (381–435)	DID-DD (135–435)
2BNX-molecule A	0.53	0.31	0.74
1Z2C	0.98	2.2	3.5
2BAP	1.5	1.9	4.8
2BNX-molocule B	0.61	0.28	10.5

The units are Å. 2BNX is the asymmetric dimeric structure of the free DID-DD-CC [34]. 1Z2C is the symmetric dimeric structure of the G-DID-DD•RhoC complex [35]. 2BAP is the symmetric dimeric structure of the DID-DD•DAD complex [40].

**Figure 3:**
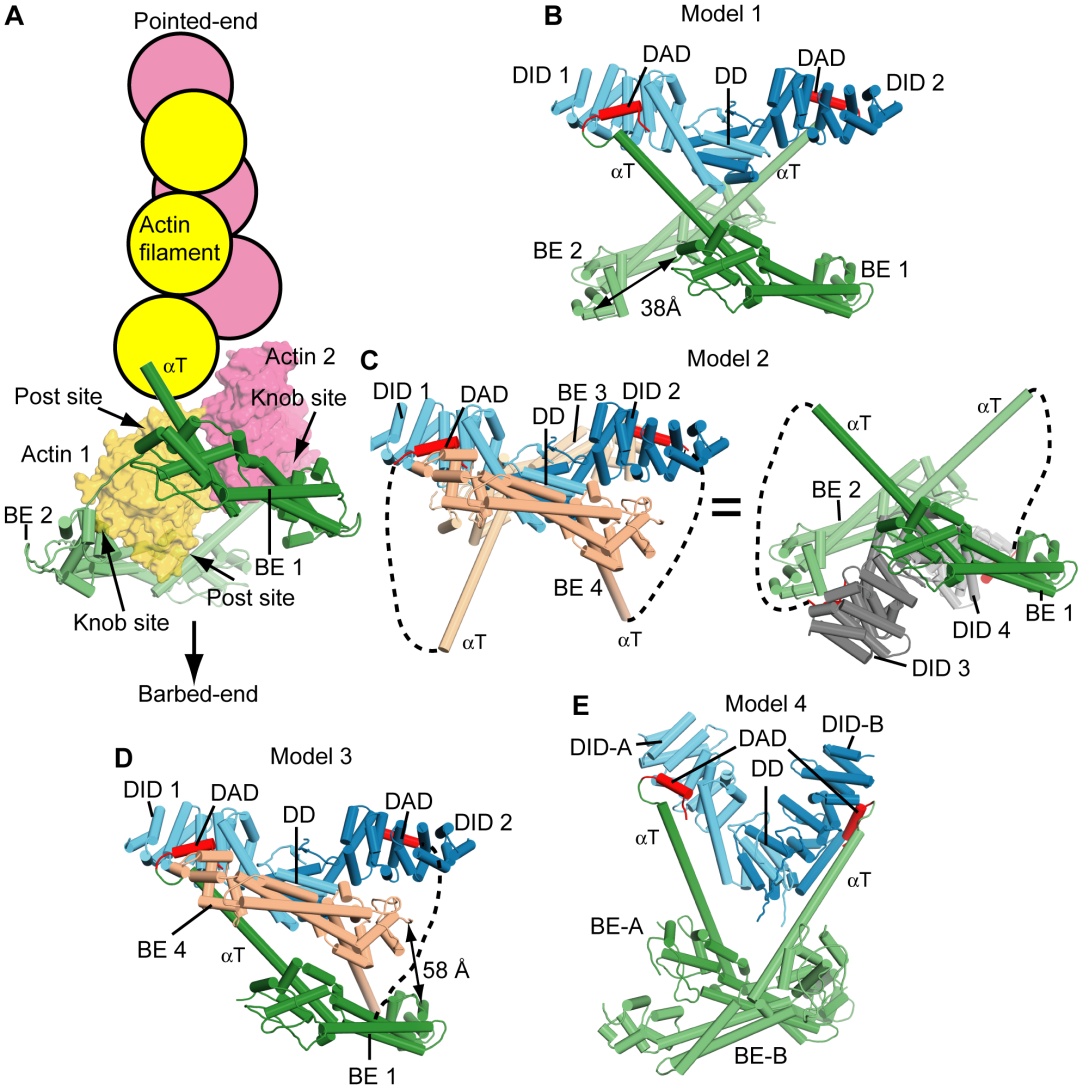
Structural models for mDia1 autoinhibition. (A) A structural model of the FH2 domain at a filament barbed-end is presented, in which a cartoon actin filament is placed at the pointed-end side of the crystal structure of Bni1p FH2 domain bound to TMR-actin (PDB code: 1Y64). (B-E) Structural models of the mDia1 (DID-DD)_2_•(FH2-DAD)_2_ dimer derived from the crystal. Dashed lines indicate linkers connecting the bridge elements and DADs. For clarity, the invisible linkers connecting two bridge elements in each model are not indicated. The names and numbering for each structural element in this figure are same as those in Figure 2 except (E) Model 4, in which two DIDs are labeled as DID-A and DID-B and the bridge elements as BE-A and BE-B. (B) In Model 1, no swapping of αT is assumed. This leads to only one bridge element bound to each DID. The dotted line is a 5 residue linker missing in the electron density map. In this configuration, the distance spanned between the two bridge elements by the lasso (∼30 residues not observed in the electron density) is 38 Å, as indicated in the figure. (C) For Model 2, two identical models are shown for clearer presentation. The model on left possess the same (DID-DD)_2_ dimer as in (B) and (D). The right is composed of the other pair from the tetramer and is to facilitate comparison with (A). The distance between the ends of dotted lines (Met1172 and Thr1179) is 73 Å, which is too long for the missing linker (5 residues) to connect. This operation would require at least the C-terminal 3∼5 turns of αT to melt. (D) Model 3 is a hybrid model between Models 1 and 2, in which αT of BE4 is swapped. The distance spanned by the missing portion of the lasso is 58 Å, as indicated in the figure. (E) In Model 4, the DID•BE pairs in Model 1 were organized according to the asymmetric structure of free DID-DD (2BNX). Thus, the relative positions within each DD-DID•DAD-BE element are the same as in (B), i.e., the position of BE-A relative to DID-A, BE-B to DID-B (Model 4), BE1 to DID1, and BE2 to DID2 (Model 1) are all the same. However, the different organization of the two DIDs in Model 1 and the asymmetric (DID-DD)_2_ dimer results in a different organization of the BEs in the two cases.

In the previously reported structures of the FH2 domains of yeast Bni1p and human Daam1 each bridge element consists of an elongated helical bundle with two helical domains at either end, termed the knob and post respectively. The bridges are connected at both ends by a so-called lasso element that extends from the knob of one bridge to wrap around the post of its partner, creating a closed ring. Binding of actin to both the knob and post sites of each bridge element is necessary for processive capping by Bni1p, and likely formins in general. A previous report described the structure of an mDia1 FH2 fragment lacking the lasso region (836–1169) [41]; the complete FH2 structure here is very similar, with backbone r.m.s.d. of 1.9 Å. The mDia1 lasso wraps around the post region as seen in Bni1p and Daam1, confirming a universal structure of the FH2 domain (Fig. 2C) [20], [42]. The backbone r.m.s.d.s between the bridge element of mDia1 and those of Bni1p and Daam1 are 3.1 Å and 4.5 Å, respectively. The most significant difference is in the knob domain, whose change alters the structural relationship between the knob and the post actin-binding sites. In the crystal structure of the Bni1p FH2-actin complex, the two actin monomers bound to each bridge element are related by a 180° rotation [21] (Fig. 3A). Although the exact 180° relationship in that system was likely enforced by crystallographic symmetry, the deviation from the ideal 166° angle in actin filaments suggested that the FH2 domain might exert strain on the filament. This, in turn, was postulated to drive fluctuations of the bridge elements necessary for processive capping [21]. The differences in the knob-post relationships in the mDia1, Bni1p and Daam1 bridge elements suggest that actin would be bound to these proteins at different angles, thus inducing different degrees of strain on filaments. This difference could play a role in governing the different rates of processive capping by various formin proteins.

The absence of electron density for the linker region (806–835) between bridge elements impairs determination of dimeric pairs of the FH2 domain in the crystal. However, the bridge elements can be connected to the DIDs with some confidence. The C-terminal α-helix of the bridge element, termed αT, extends away from the post domain for ∼10 turns (Fig. 2C). At Leu1169 it connects to a loop that joins to the α-helical region of the DAD (Val1181-Gly1192), which is bound to the concave surface of the DID (Fig. 2D) and ends at Phe1195. The cluster of basic residues after Phe1195, which contributes to high affinity between the N- and C-termini [43], is not clearly observed in our electron density map. The weak electron density for the αT-DAD loop suggests that this element is flexible. The interaction between the DID and DAD is essentially the same as observed in the previously described DID•DAD and DID-DD•DAD complexes with backbone r.m.s.d. of 0.6 Å and 1.5 Å, respectively, between DAD on superimposed DIDs [36], [40].

The asymmetric unit contains four chains of the DID-DD and bridge-DAD proteins, which form a tetrameric configuration (Fig. 2A). In this arrangement, the two actin-binding sites on each bridge element face toward the center of the structure, where there is no space for actin monomers (compare Figs. 2A and 3A). The residues important for actin-binding make contacts with other parts of the molecules (Fig. S3). Ile845, a critical residue in the knob site [20], [21], [41], makes contacts with aliphatic chains of Gln1161, Gln1164, Lys1165 and Gln1168 from αT of another bridge element. The positively charged residues in the post site involving the lasso (Arg769, Arg764, and Arg975) are positioned adjacent to a highly negatively charged patch on the DID located at its convex surface toward the end of the long axis (Glu227, Glu229, Glu230, Glu268, Glu272, Glu276, and Glu279). The DD also contacts a region adjacent to the post site between two actin-binding sites. Thus, the structure suggests that this tetrameric form of the complex would be inhibited for nucleation and processive capping due to an inability to bind actin monomers and filaments. This mechanism is analogous to that seen in many autoinhibited systems, where a regulatory domain binds directly to the active site of a functional domain, blocking its interactions with substrates/ligands [39].

In the crystal, the tetramer makes contacts with other tetramers through interactions between the (DID-DD)_2_ dimers. On the basis of this interaction it is possible to generate two alternative tetrameric configurations in addition to that shown in Figure 2A (Fig. S4). In these configurations, the tetramer consists of a pair of (DID-DD)_2_•(bridge-DAD)_2_ dimers held together by contacts between the (DID-DD)_2_ elements. Inhibition in these systems would occur through the mechanisms described below for potential mDia1 dimers. In these alternative tetramers, dissociation of (DID-DD)_2_-(DID-DD)_2_ contacts could explain the dimer-tetramer equilibrium observed in the analytical ultracentrifugation and MALS data. Regardless of exactly which tetramer (or combinations thereof) predominates in solution, the domain-domain contacts within mDia1 are likely to be quite dynamic, since the system can generate the intertwined tetramer in the crystal as described above. Indeed the lasso-post interface was swapped during crystallization of the yeast FH2-actin complex in a previous study [21], suggesting that domain-domain contacts may be generally dynamic in formins.

### Possible Autoinhibitory Mechanisms of an mDia1 Dimer

Although the structure of the (DID-DD)_4_•(bridge-DAD)_4_ tetramer explains clearly why it is inactive, full length mDia1 contains only two copies of each element. Can the tetramer structure suggest mechanisms of autoinhibition in the intact protein? Although the dimeric pairing of bridge elements to produce the functional FH2 domain cannot be definitively determined in our structure, it is possible to extract (DID-DD)_2_•(bridge-DAD)_2_ complexes from the tetramer. Below, we describe four possible structures and their relevance to autoinhibition in the mDia1 dimer (Fig. 3). A common assumption in all of the models is that the (DD)_2_ dimer, and therefore the DID-DID pairing, observed in the crystal is representative of the N-terminal dimer in the full-length protein. This assumption is based on the high homotypic affinity in the dimeric N-terminus [32] and the high structural similarity between the (DD)_2_ domain in the tetramer here and the three previously reported structures of the mDia1 N-terminus[34], [35], [40].

For the first model, the (DID-DD)_2_ pair is coupled to the two bridge-DAD elements specified by the DID•DAD interaction in the tetramer (Model 1, Fig. 3B). In this model, the (DID-DD)_2_ dimer sits over the bridge elements in the pointed-end direction (compare Figs. 3A and 3B). Here, the bridges have no contact with any other part of the complex, and therefore the two actin-binding sites on each bridge are exposed to solvent. Nevertheless, if the crystallographic orientation were rigidly adopted, the two bridge elements would be positioned too closely to allow binding of actin monomers (Fig. S5A). However, such rigidity is unlikely given the known flexibility of the bridge-bridge and αT-DAD linkers, and the likely fluctuations of αT in solution. In fact, we found that introducing flexibility in αT by replacing a portion of the helix with a Gly-Gly-Ser linker or by extension of the αT-DAD linker, did not affect inhibition by G-DID-DD-CC (Fig. S6), indicating that the rigidity of αT is not an important factor for autoinhibition. This model thus suggests that one or two actin monomers might be able to bind to bridge elements of the autoinhibited protein (see also below). However, even with flexibility in the structure, comparison of Figures 3A and 3B suggests that the (DID-DD)_2_ element would sterically clash with additional monomers that attempted to extend a filament beyond this initial nucleus. This suggests that autoinhibition may arise because of steric occlusion of an actin filament by the (DID-DD)_2_ dimer. (Note that the activities of the FH2 domain, i.e., *de novo* nucleation of filaments and processive capping/elongation, both require filament binding. Thus blocking filament binding in the autoinhibited state should lead to inhibition.) This is consistent with previously reported biochemical data showing that autoinhibited mDia1 does not bind filaments in an actin sedimentation assay [44].

An alternative organization of the full-length dimer would involve binding of the bridge pair in Model 1 to the other (DID-DD)_2_ dimer (or equivalently binding of the (DID-DD)_2_ dimer in Model 1 to the other bridge pair) (Fig. 3C, Model 2). This interaction would invoke a DID-DAD domain swap during crystallization, and would require melting of αT. The feasibility of such operations is supported by our observation that increased flexibility of αT does not affect inhibition by G-DID-DD-CC (Fig. S6). In this model the two DIDs sit between the bridge elements, and occlude both of the actin-binding sites as in the tetramer. In this case, there are physical contacts between DID-DD and bridge elements as described above for the tetramer (Fig. S3). However, we failed to demonstrate interactions between DID-DD and bridge element in solution NMR experiments. The ^1^H-^15^N TROSY-HSQC spectra of 250 µM ^2^H/^15^N-labeled DID complexed with unlabeled DAD in the absence and presence of unlabeled DAD-less FH2 (250 µM) shows no peak shifts or broadening (Fig. S7), indicating that there is no interaction between DID and bridge at this concentration range (or the population of the DID-bridge complex is too small to detect by this method). Thus Model 2 is unlikely to be a highly populated structure in the autoinhibited state of full-length mDia1.

Another possible model (Model 3, Fig. 3D) is comprised of the (DID-DD)_2_ dimer in Model 1 and another pair of the bridge-DAD (BE1 and BE4). This configuration also requires melting αT of BE4 to make the DID-DAD contacts as similarly discussed for Model 2 above. Actin binding to BE4 is impaired by the described physical contacts for the knob and post actin-binding sites. Actin monomer should be accessible to the post site of BE1 but not to the knob site due to steric clash of actin with BE4 (Fig. S5B). These considerations suggest that Model 3 is also inhibited by the incompatibility with actin monomer and filament binding.

One final possibility is that in the presence of the CC (or in the full-length protein) the organization of the N- and C-termini is different from any potential model created from the tetrameric crystal structure. Specifically, since the CC element appears to induce an asymmetric structure in the (DID-DD-CC)_2_ dimer [34], it is possible that in the autoinhibited full-length protein the N-terminus adopts this same asymmetric conformation as well. In Figure 3E (Model 4) we have modeled the two bridge-DAD elements from the tetramer structure onto the asymmetric (DID-DD-CC)_2_ dimer (PDB: 2BNX) using the DID•DAD interactions in the tetramer. In Model 4, the relative position and orientation of the two bridge elements are completely different from that in the tetrameric crystal structure. However, a similar mechanism of autoinhibition emerges here as for Model 1. If the structure were rigid, steric clashes would occur between actin monomers on one bridge and either the neighboring bridge or the DID-DD, depending on the binding site engaged (Figs. S5C and S5D). If the structure were flexible (as is more likely), the overall configuration would permit binding of the initial actin monomers, but is incompatible with addition of further monomers to produce a filament.

The four models presented here would respond to Rho GTPases differently. Previous studies of mDia1 have shown that Rho binds to the G-DID element competitively with the DAD, and thus would cause dissociation of these intramolecular contacts in the full length protein. In Models 1 and 4 one molecule of Rho would be sufficient for activation, because disruption of a single DID-DAD contact would enable the DID-DD element to swing away from its position over the FH2 ring, allowing processive capping to occur. In the case of Model 2, however, two molecules of Rho would be required to expose both of the actin binding sites that are covered by the (DID-DD)_2_ dimer. Since Model 3 is a hybrid of Models 1 and 2, depending on which DID•DAD contact Rho disrupts, the number of Rho molecules could be one (when Rho binds to DID 2) or two (when Rho binds to DID 1).

### Binding of Monomeric Actin to the Autoinhibited Complex

**Figure 4:**
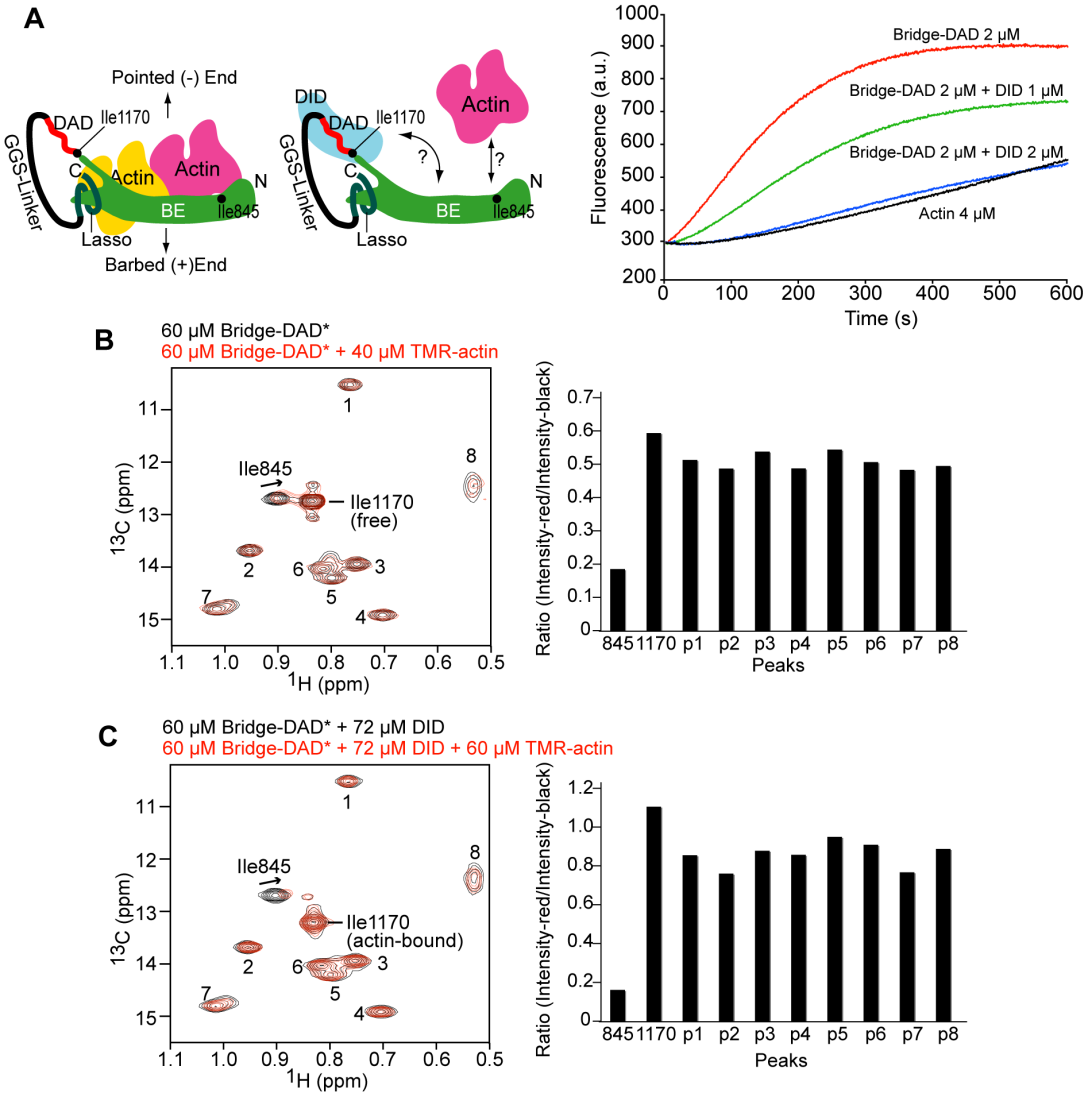
BE-DAD is inhibited by DID but actin monomer is accessible to the knob site. (A) Pyrene actin assembly assays indicating that DID inhibits BE-DAD-stimulated actin assembly. A cartoon representing the artificial BE-DAD construct is shown on the left. (B, C) ^1^H-^13^C Ile δ1 methyl-TROSY HMQC spectra of 60 µM BE-DAD in the absence (B) or presence (C) of 72 µM DID are shown in black. The spectra in the presence of TMR actin are shown in red. Asterisk indicates the isotope-labeled material (BE-DAD) and others are unlabeled. In both (B) and (C), addition of TMR actin causes broadening and small shifts of the Ile845 resonance, indicating binding. Ile845 is located in the center of the knob actin binding site (Fig. 2C and refs. [20], [21]). The Ile1170 peak shifts upon addition of DID to BE-DAD (from (B) to (C)) because Ile1170 contacts the DID as shown in Fig. 2D. Ratios of the intensities of red/black peaks are plotted for each peak on the right panels. The ratios for the Ile845 peaks are significantly smaller than the other peaks in both (B) and (C), strongly suggesting that Ile845 interacts with actin.

The common and most likely mechanism derived from the above considerations invokes inhibition of filament binding by the dimeric DID-DD N-terminus when tethered to the two DAD elements emanating from the FH2 dimer. Since DID does not contact FH2 directly, dimerization of the N-terminus is necessary for inhibition, as a DID monomer bound to DAD would not be constrained into the inhibitory configuration over the FH2 ring. However, Li and Higgs have shown that a DID monomer can inhibit FH2-DAD [32]. In that study, the concentration for half inhibition by the DID monomer was about 120 nM, which is consistent with the reported DID-DAD affinity (K_D_ = 100∼250 nM) [32], [36], [40]. These data suggest that the DID-DAD interaction alone governs the inhibition without any need for DID dimerization. To resolve this apparent conflict between structural and biochemical data, we examined whether the inhibitory action of monomeric DID is limited to the FH2 dimer (two bridges) or if it could also block the activities of an engineered monomeric bridge element [21]. In a standard actin assembly assay, the monomeric bridge element nucleates filaments that grow slowly at their pointed-ends while the barbed-ends are blocked [20], [21]. As shown in Figure 4A, we found that stoichiometric concentration of a DID monomer (2 µM) inhibits this activity of bridge-DAD (2 µM). These data led us to consider a possibility in which bridge and DID may be joined through an actin monomer in a way that inhibits filament nucleation or growth. This mechanism would be consistent also with the dimer models presented above: actin monomer could be incorporated into the dimer models because the actin-binding sites are not occluded and the dimeric architectures are likely flexible in solution.

To begin testing this hypothesis, we took advantage of methyl-TROSY NMR experiments to examine binding of the mDia1 FH2 domain to monomeric actin [21]. The δ1 methyl resonances of Ile845, located in the knob actin-binding site, and Ile1170, located at the C-terminal end of αT (Fig. 2D) are very intense compared to the other isoleucine resonances in the bridge-DAD protein (Fig. 4B, resonances assigned by mutagenesis, see Methods). These are the only two isoleucine sidechains in the protein that are exposed to solvent. The Ile845 resonance is significantly broadened upon addition of tetramethylrhodamine (TMR)-actin compared to other signals in the spectrum (Fig. 4B), likely due to a combination of chemical exchange and degradation of the TROSY effect by protons on actin [21]. In contrast to TMR-actin, addition of unlabeled DID to bridge-DAD did not cause significant broadening of bridge resonances except Ile1170 (Figs. 4C and S8). The relative sharpness and unchanged peak position (^1^H and ^13^C chemical shifts) of the Ile845 resonance suggests that Ile845 remains solvent-exposed in the DID complex with bridge-DAD. The shift and the broadening in the Ile1170 resonance (again, assigned by mutagenesis of the complex) upon binding to DID (Figs. 4B, 4C and S8) are consistent with contacts of Ile1170 to the DID in the crystal structure of the tetramer (Fig. 2D). When TMR-actin was added to the complex, the Ile845 resonance was broadened appreciably more than others (Fig. 4C), indicating that actin monomer interacts with the knob site, even in the presence of DID. Thus inhibition in the complex between bridge-DAD and DID does not occur through occlusion of both actin-binding sites, and could potentially involve a sandwich structure between DID, actin and bridge-DAD. We have no information on actin binding at the post site due to lack of an appropriate NMR probe at this location of the molecule. Analogous experiments to examine binding of actin to the knob site of the (G-DID-DD-CC)_2_•(bridge-DAD)_2_ were inconclusive due to the large size of the complex (221 kDa) and consequent poor quality of the NMR data. Nevertheless, the accessibility of actin-binding sites in the models of full-length mDia1 presented above (Fig. 3), coupled with the ability of actin monomer to bind to the inhibited DID•bridge-DAD system (Fig. 4) suggests that autoinhibition may involve not only DID•bridge-DAD complexes, but also actin monomer as well.

### Conclusion

We have discussed several possible mechanisms of autoinhibition of mDia1 based on our initial structure and proposed that structural hindrance between actin filament and the bridge elements, which occurs in the geometry of the autoinhibitory state, is the likely one. Based on additional biochemical and NMR data we also suggest the possibility that autoinhibition in full-length mDia1 may involve actin monomer binding. Assessment of the presented possible mechanisms will require structure determination of larger mDia1 constructs in future.

## Materials and Methods

### Protein preparation

The cDNA clone for mouse mDia1 (ID: 5042572, GI:47124598) was purchased from Invitrogen and used as a PCR template to subclone the various fragments used here. For the oligomerization analyses of intact mDia1 protein, we used a construct harboring residues 67–1209, which contains all of functional domains in mDia1 (see Fig 1A; the yield of the complete full-length mDia1 (1–1255) was very poor in *E.coli* and insect cells). For simplicity, this slightly truncated construct is referred to as the “full-length” protein throughout the text. mDia1 full-length (67–1209) was expressed in Sf9 (Spodoptera frugiperda) insect cells (Invitrogen) by infecting with baculovirus carrying the mDia1 cDNA. The baculovirus was generated using Bac-to-Bac Baculovirus Expression System (Invitrogen) with a modified pFastBac HTa plasmid (Invitrogen) carrying mDia1 (67–1209) at the NdeI-XhoI sites (NdeI and a thrombin recognition sequence for His-tag cleavage had been incorporated into the original pFastBac HTa by linker ligation). The expressed Hig-tagged protein was purified with Ni-Sepharose FF (GE healthcare) followed by MonoQ anion exchange and Superdex 200 gel filtration columns. The His-tag was cleaved off by thrombin (GE healthcare) before gel filtration.

The following pET vectors (Novagen) harboring mDia1 fragments at NdeI-XhoI sites were generated using standard cloning procedures: pET32b for G-DID-DD-CC (residues 67–570), pET11a for DID-DD (131–457), DID (131–370), FH2 (753–1159) and FH2-DAD (753–1209). The genes for the following artificial mDia1 fragments were generated by multiple PCR reactions and cloned into the NdeI-XhoI sites of pET vectors: pET41a for bridge-DAD (828-1209+15 repeats of (Gly-Gly-Ser) + Ala-Met + 753-813), FH2-GGS20r-DAD (753–1151+6 repeats of (Gly-Gly-Ser) + Gly-Gly + 1172–1209) and FH2-GGS30i-DAD (753–1159+10 repeats of (Gly-Gly-Ser) + 1160–1209), or pET11a for FH2-GGS12r-DAD (753–1159+4 repeats of (Gly-Gly-Ser) + 1172–1209). Proteins were expressed in *E.coli* BL21(DE3) at 20°C overnight. None of these proteins contained any affinity tags; all were purified using standard chromatographic columns: DEAE sepharose, MonoQ, MonoS and Superdex 200 gel filtration columns for the N-terminal fragments, and SP sepharose, MonoS and Superdex 200 gel filtration columns for the C-terminal fragments. The isoleucine δ1-protonated, otherwise deuterated proteins for methyl-TROSY NMR experiments were produced using D_2_O M9 minimal media containing alpha-ketobutyric acid (Isotec, USA) as a precursor for isoleucine as described [45]. Actin was prepared from rabbit muscle and labeled with tetramethylrhodamine as described previously [46], [47].

### Pyrene actin assembly assays

Actin assembly assays using 5% pyrene actin were performed as previously reported [48].

### Determination of oligomerization states of mDia1 proteins

All samples used for determination of oligomerization were passed through a gel filtration column to assure that there was no aggregate, and to remove any excess of the N- or C-terminal fragment from 1∶1 stoichiometric complex. Sedimentation equilibrium experiments were performed for the mDia1 autoinhibited complexes (DID-DD•FH2-DAD and G-DID-DD-CC•FH2-DAD) and the full-length protein (67–1209) using a Beckman XL-A analytical ultracentrifuge with the four-position An60Ti rotor. Protein complex solutions were loaded at initial concentrations of 7, 14, and 28 µM in 10 mM HEPES pH 7.0, 150 mM NaCl, 0.5 mM DTT. Data of absorbance at 280 nm were acquired at three different speeds (6500, 9500, and 12000 rpm) at 20°C and analyzed using a nonlinear least-square curve fitting program Winnonln (Written by David Yphantis). Protein partial specific volume and buffer viscosity were determined using Sednterp (program written by David Hayes and Tom Laue). MALS experiments of the wild-type FH2 domain of mDia1 autoinhibited complexes were performed with on-line light scattering instrument connected with a size exclusion column. 200 µl of 25 µM mDia1 proteins were injected into Superdex 200 gel filtration column (GE healthcare) equilibrated with 10 mM HEPES pH 7.0, 150 mM NaCl 1 mM DTT. The light scattering was monitored with an 18-angle light scattering detector (DAWN EOS) and refractive index detector (Optilab DSP) (Wyatt Technology). Data were collected every 0.5 s at a flow rate of 0.4 ml/min. Data analysis was carried out using the program ASTRA version 4.90 (Wyatt Technology), yielding the molar mass and mass distribution of the samples. No band broadening correction was applied due to unavailability of this function in this version of the ASTRA software. However, our data generate reasonably flat molar masses, indicating that the band broadening effect due to dilution between light scattering and RI detectors was very small and not large enough to change our interpretation of the oligomerization states of the mDia1 proteins.

### Crystallization and X-ray diffraction data collection of the DID-DD•FH2-DAD complex

Crystals of the mDia1 DID-DD (131–457)•FH2-DAD (753–1209) complex in 10 mM HEPES pH 7.0, 150 mM NaCl, 1 mM DTT were grown at 20°C using the hanging-drop vapor diffusion method from drops containing 1 µl protein (30 mg/ml) and 1 µl of reservoir solution (100 mM MES pH 6.75, 12% (w/v) PEG1500). Needle-shaped crystals appeared immediately upon setup and grew to several hundred µm in one dimension but not in the other dimensions. These crystals were used for streak seeding into fresh drops which had been pre-equilibrated versus 100 mM MES pH 6.75, 10% (w/v) PEG1500, 25% sucrose for 24 hours before seeding. Single crystals grew in this condition to 300×100×60 µm in one week, and were cryo-protected with 100 mM MES, pH 6.75, 11.5% (w/v) PEG1500, 150 mM NaCl, 35% (v/v) sucrose, and flash-cooled in liquid propane. All crystals (native and selenomethionyl-substituted) were grown and cryoprotected in a similar manner, and exhibit the symmetry of space group *P2_1_* with cell dimensions of *a* = 93.9 Å, *b* = 208.5 Å, *c* = 131.5 Å, β = 102.7° and contain four DID-DD and four FH2-DAD molecules in the asymmetric unit. Native crystals diffracted to a d_min_ of 2.75 Å when exposed to synchrotron radiation. Crystals of a complex of selenomethionyl-substituted mDia1 DID-DD (131–457) and native mDia1 FH2-DAD (753–1209) (SeMet #1) diffracted to a d_min_ of 2.95 Å when exposed to synchrotron radiation. Crystals of a complex of native mDia1 DID-DD (131–457) and selenomethionyl-substituted mDia1 FH2-DAD (753–1209) (SeMet #2) diffracted to a d_min_ of 3.45 Å when exposed to synchrotron radiation. All diffraction data were collected at beamline 19-ID (SBC-CAT) at the Advanced Photon Source (Argonne National Laboratory, Argonne, Illinois, USA). Data were indexed, integrated and scaled using the HKL-2000 program package [49]. Data collection statistics are provided in Table 1.

### Phase determination and structure refinement

Initial phases for the native mDia1 DID-DD (131-457)•FH2-DAD (753-1209) complex were obtained via molecular replacement in the program AMoRe [50] using a truncated version of chain A of the previously determined structure of the mDia1 DID-DD-CC (131–516) domain (PDB ID 2BNX) [34]. The molecular replacement solution was verified and 60 selenium sites were identified via an anomalous difference map using data collected at the peak wavelength for selenium from the SeMet #1 crystals. Phases for a two-wavelength selenium anomalous dispersion experiment with SeMet #1 crystal data to a d_min_ of 2.95 Å were refined with the program MLPHARE [51], resulting in an overall figure-of-merit of 0.20 for data between 35.0 and 2.95 Å. Phases were further improved by four-fold averaging and density modification with the program DM [52] resulting in a figure-of-merit of 0.44. Clear density was seen in the map for the C-terminal bridge element of mDia1. Manual placement and rebuilding of a model for the mDia1 bridge element using the previously determined structure of the core FH2 domain of mDia1 [41] (PDB ID 1V9D) were performed in the program O [53]. Confirmation of the model building for the bridge element was obtained via inspection of an anomalous difference map using data collected at the peak wavelength for selenium from the SeMet #2 crystals. Refinement was performed with the native data to a resolution of 2.75 Å using the program PHENIX [54] with a random 1.5% of all data set aside for an R_free_ calculation. The final model contains four mDia1 DID-DD (131–457)•FH2-DAD (753–1209) complexes; included are residues 131–452, 754–805, 829–1195 of complex A; 132–192, 200–457, 754–805, 830–1196 of complex B; 131–452, 754–805, 830–1196 of complex C; and 131–457, 754–805, 830–1196 of complex D. The R_work_ is 0.199, and the R_free_ is 0.261. A Ramachandran plot generated with Molprobity [55] indicated that 94.1% of all protein residues are in the most favored regions.

### Methyl-TROSY NMR experiment

Isoleucine δ1 methyl-protonation in the otherwise deuterated background was employed for the bridge-DAD protein for methyl TROSY HMQC experiments. 60 µM labeled bridge-DAD was dissolved in the NMR sample buffer containing 10 mM Hepes, pH7.0, 1 mM DTT, 100 mM KCl, 100 µM CaCl_2_, 100% D_2_O. TMR-actin was stored at 400 µM protein concentration in 2 mM Tris pH7.0, 100 µM CaCl_2_, 1 mM EGTA, 1 mM NaN_3_, 100% D_2_O until use. When TMR-actin was added the NMR sample, salts were also added to maintain the total concentration at 100 mM. ^1^H-^13^C methyl-TROSY HMQC spectra were recorded at 25°C using 800 MHz Varian INOVA NMR equipped with the cryogenic triple resonance probe. Experimental time for each spectrum was 4∼8 hours. Data processing and analysis were carried out using NMRPipe and NMRDraw [56] and the figure was generated using NMRView [57].

## Supporting Information

Figure S1 Deletion of the CC does not affect the oligomerization state of the N-terminus. MALS data for (A) G-DID-DD-CC and (B) DID-DD were obtained and analyzed as described in Materials and Methods. The molar masses indicate that both proteins are dimers in solution.
(0.35 MB TIF)

Figure S2 DID-DD inhibits FH2-DAD as potently as G-DID-DD-CC. 5 µM FH2-DAD was completely inhibited by 10 µM of (A) the G-DID-DD-CC dimer or (B) the DID-CC dimer in actin assembly assays.
(0.34 MB TIF)

Figure S3 Molecular contacts at the Knob and Post sites of bridge elements. The view of this figure is roughly from the right bottom of the right panel of Fig. 2A to DID1. The residues mentioned in the text are shown as sticks.
(2.68 MB TIF)

Figure S4 Alternative tetramers based on crystallographic contacts. (A) Two crystallographic asymmetric units are shown. The contacts between two (DID-DD)4•(FH2-DAD)4 tetramers are indicated by purple circles. The top and bottom tetramers correspond to the complex in Figure 2A. (B, C) Alternative tetramers extracted from (A) are shown. In (B) two of the Model 1 dimer form a tetramer through indicated contacts. Similarly in (C) two of the Model 2 dimer form a distinct tetramer. (D) An expanded view of the crystallographic contacts. These contacts involve residues immediately following the DD (in what would be the DD-CC linker) and residues from the loops between α helices in the armadillo structure of the DID. Nomenclature for the indicated helices is from the previous work [34]. The two DD extensions (residues 446-451) in the DID-DD dimer are in extended conformation and aligned anti-parallel to each other. (E) The structure of the free DID-DD-CC (PDB code: 2BNX) shows a parallel configuration of the DD-CC linkers in the dimer, presenting entirely different molecular surface at the site of the crystallographic contacts discussed illustrated in panel D. This difference in structure may explain why DID-DD-CC constructs produce dimers while DID-DD constructs produce dimer-tetramer mixtures.
(3.88 MB TIF)

Figure S5 Demonstration of compatibility of actin binding to Models 1 and 4. Actin was modeled onto (A) BE1 of Model 1, (B) BE1 of Model 3, (C) BE-B of Model 4, and (D) BE-A of Model 4 (C) by superimposing the structure of the Bni1p bridge element bound to two actin molecules (PDB code; 1Y64). Actin 1 in yellow is bound to the post site of the Bni1p BE and Actin 2 in pink to the knob site. Actin 1 and 2 are related by pseudo two fold symmetry [21]). Observed steric clashes with actin are indicated by red colored parts of mDia1. DAD is not colored in red only in this figure for clarity. In (A), major clashes occur between both actin molecules and the other bridge (BE2) but not with DID-DD. In (B), Actin 2 clashes with BE4. In (C), Actin 2 but not Actin 1 clashes with DD. In (D), Actin 2 clashes with BE-A but not with DID-DD and CC potentially clashes with Actin 1.
(2.84 MB TIF)

Figure S6 Introducing flexibility in αT does not affect inhibition by the N-terminus. Inhibition of (A) FH2-DAD wild type or (B-D) artificial constructs by the G-DID-DD-CC dimer was tested in actin assembly assays. In (B), FH2-GGS12r-DAD, in which 12 residues in αT (1160–1171) were replaced with 4 repeats of Gly-Gly-Ser sequence, shows actin assembly activity at a similar level as wild type and can be inhibited. In (C), FH2-GGS20r-DAD including replacement of 20 residues (1152–1171) shows slightly less activity and can also be inhibited. In (D), FH2-GGS30i-DAD, in which 10 repeats of Gly-Gly-Ser sequence was inserted between 1159-1160, shows similar activity and can also be inhibited.
(0.66 MB TIF)

Figure S7 Isolated DID•DAD complex does not interact with the FH2 domain. An overlay of the 1H/15N HSQC spectra of the complex of 15N-DID bound to unlabeled DAD shows no significant changes in peak position or linewidth upon addition of unlabeled FH2 domain (lacking DAD). Because of the high molecular weight of the FH2 domain, even weak interactions should have caused observable peak broadening. Thus it is very likely that the FH2 domain does not interact with DID at a measurable affinity when it is not tethered to the N-terminus through the linked DAD.
(0.72 MB TIF)

Figure S8 Peak intensities in the 1H-13C Ile δ1 methyl-TROSY HMQC spectra of BE-DAD in Figure 4. Raw peak intensities of the spectra of BE-DAD in the (A) absence or (B) presence of unlabeled DID (both in the absence of TMR-actin and shown in black in Figs. 4B and 4C). In (C), the ratios of the intensities in (B) divided by those in (A) for each peak are shown. The higher intensity of the Ile1170 resonance in (A) indicates that the DAD region is highly mobile in the free BE-DAD protein. In the absence of actin, the Ile845 resonance is affected by DID to the same extent as the unassigned peaks 1-8 (which represent residues in the core of the BE), while the Ile1170 resonance is greatly decreased on intensity (panel C). This is consistent with the DID differentially decreasing the mobility of Ile1170 in the DAD region. This behavior contrasts with that shown in Figures 4B and C, where actin differentially broadens the Ile845 resonance.
(0.28 MB TIF)
