# Supplementary figures and images for: Crystal Structure of the Formin mDia1 in Autoinhibited Conformation
Source: PLoS One. 2010 Sep 30;5(9):e12896. doi: 10.1371/journal.pone.0012896 (PMC2948019; doi:10.1371/journal.pone.0012896)

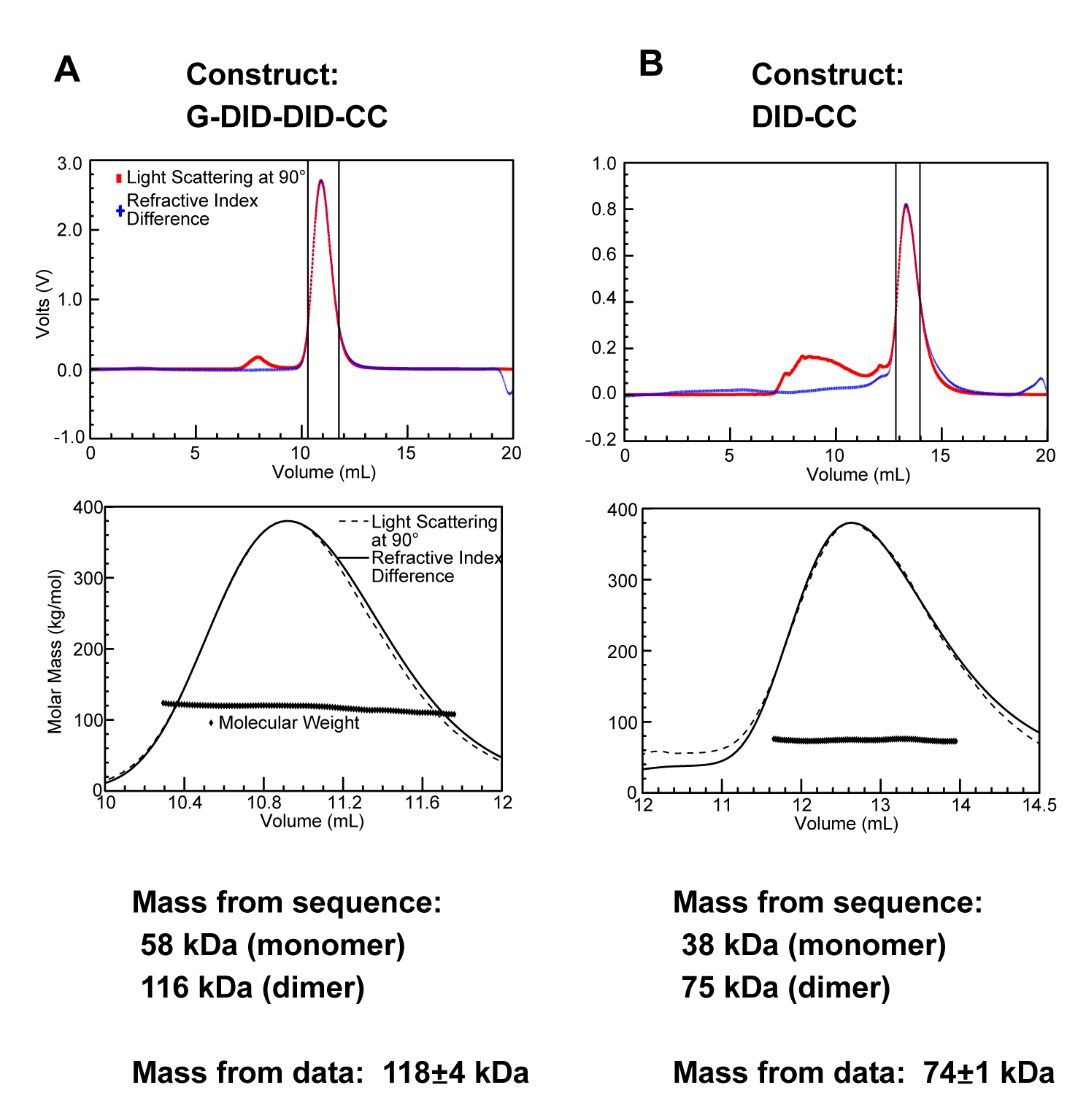

Supplement: Figure S1 — Deletion of the CC does not affect the oligomerization state of the N-terminus. MALS data for (A) G-DID-DD-CC and (B) DID-DD were obtained and analyzed as described in Materials and Methods. The molar masses indicate that both proteins are dimers in solution. (0.35 MB TIF) [file pone.0012896.s001.tif]

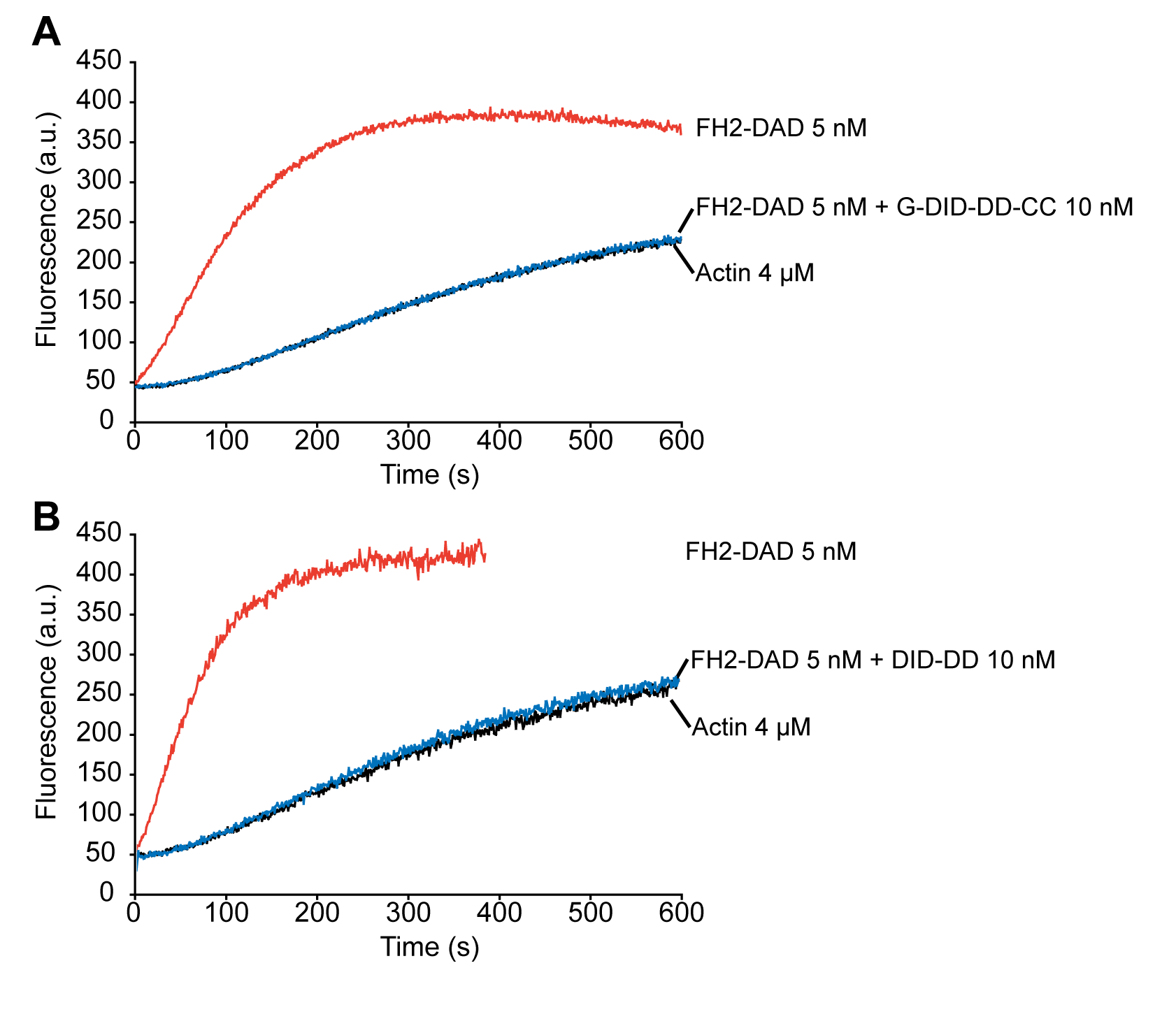

Supplement: Figure S2 — DID-DD inhibits FH2-DAD as potently as G-DID-DD-CC. 5 µM FH2-DAD was completely inhibited by 10 µM of (A) the G-DID-DD-CC dimer or (B) the DID-CC dimer in actin assembly assays. (0.34 MB TIF) [file pone.0012896.s002.tif]

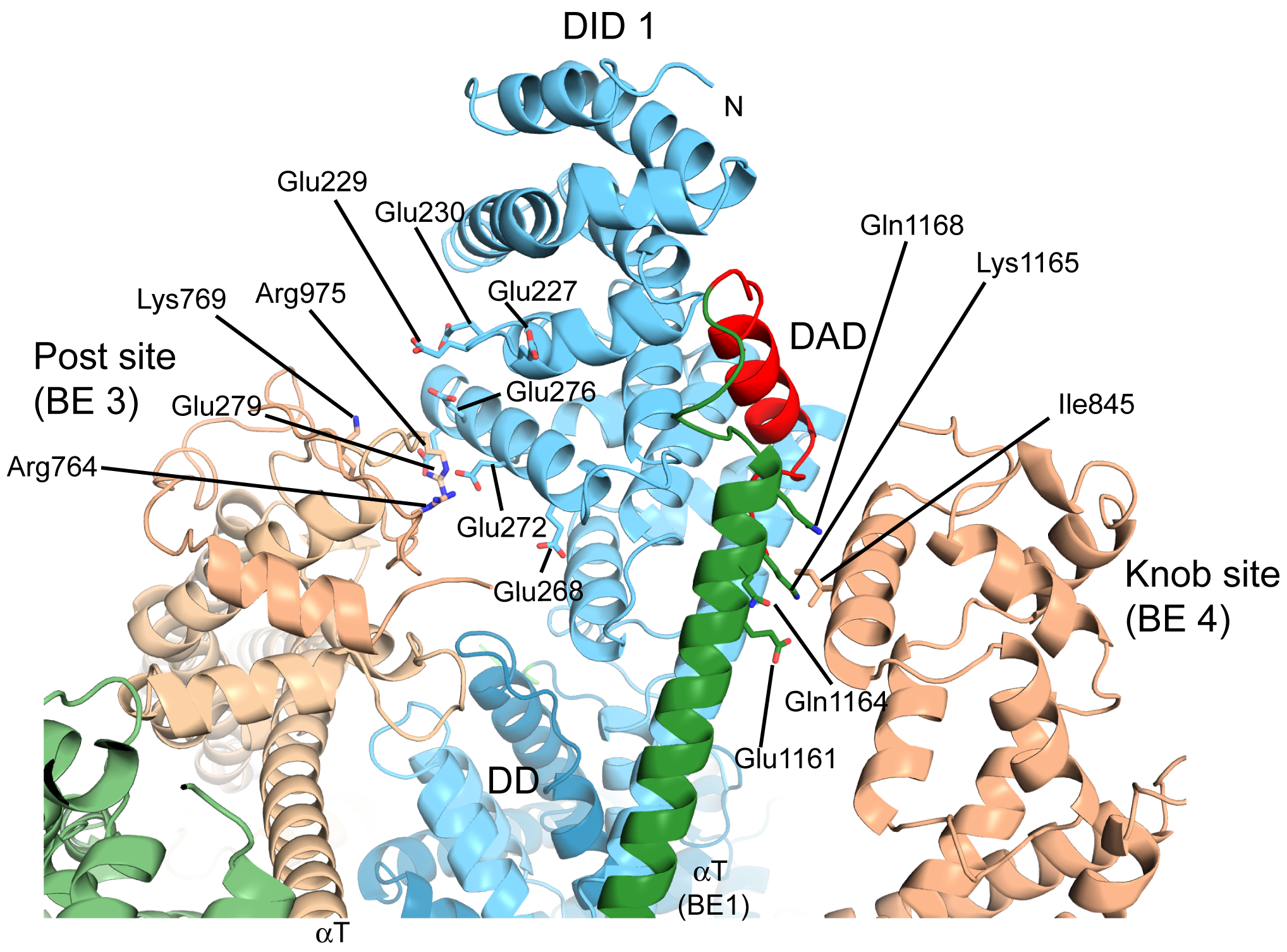

Supplement: Figure S3 — Molecular contacts at the Knob and Post sites of bridge elements. The view of this figure is roughly from the right bottom of the right panel of Fig. 2A to DID1. The residues mentioned in the text are shown as sticks. (2.68 MB TIF) [file pone.0012896.s003.tif]

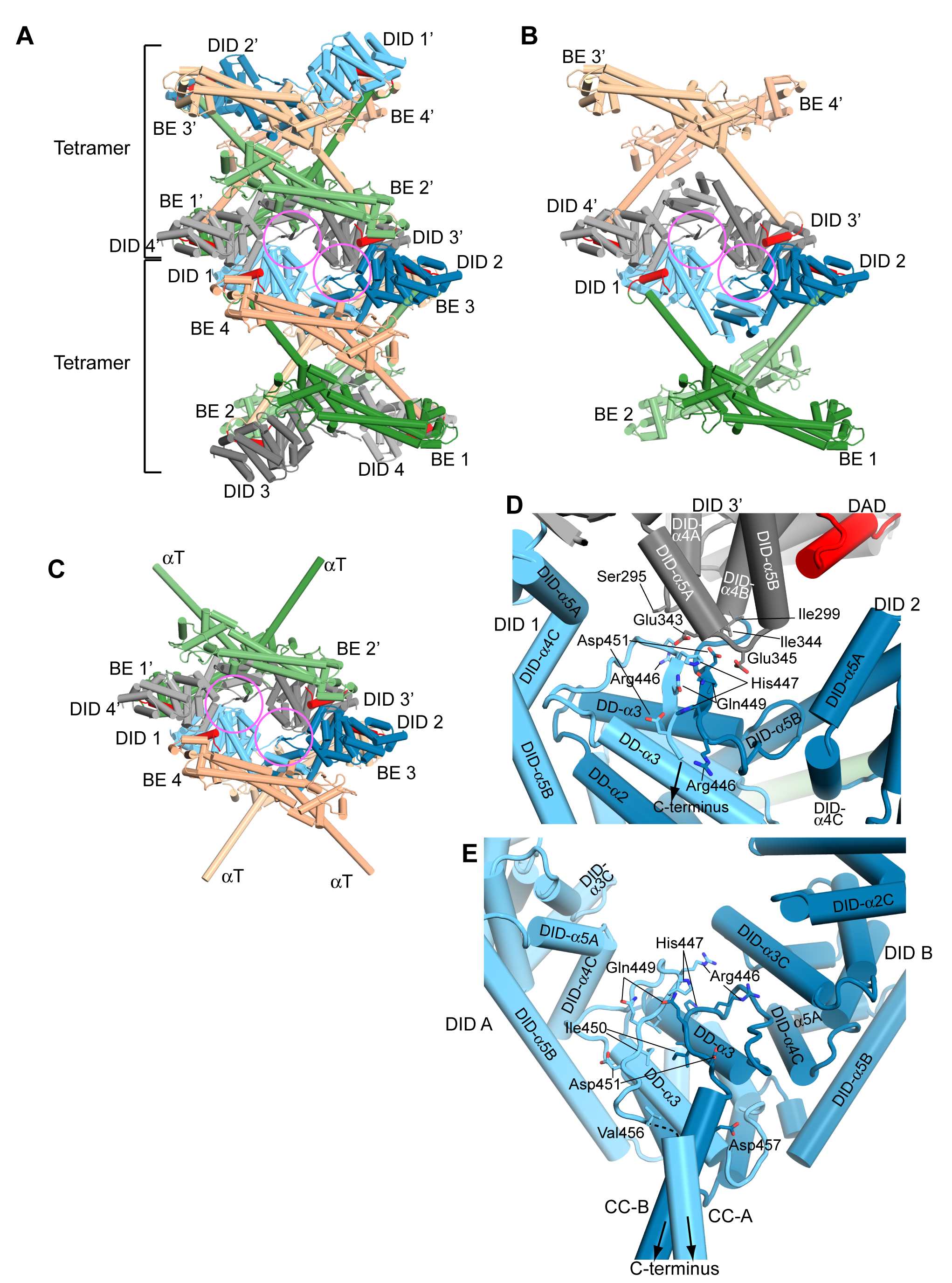

Supplement: Figure S4 — Alternative tetramers based on crystallographic contacts. (A) Two crystallographic asymmetric units are shown. The contacts between two (DID-DD)4•(FH2-DAD)4 tetramers are indicated by purple circles. The top and bottom tetramers correspond to the complex in Figure 2A. (B, C) Alternative tetramers extracted from (A) are shown. In (B) two of the Model 1 dimer form a tetramer through indicated contacts. Similarly in (C) two of the Model 2 dimer form a distinct tetramer. (D) An expanded view of the crystallographic contacts. These contacts involve residues immediately following the DD (in what would be the DD-CC linker) and residues from the loops between α helices in the armadillo structure of the DID. Nomenclature for the indicated helices is from the previous work [34]. The two DD extensions (residues 446-451) in the DID-DD dimer are in extended conformation and aligned anti-parallel to each other. (E) The structure of the free DID-DD-CC (PDB code: 2BNX) shows a parallel configuration of the DD-CC linkers in the dimer, presenting entirely different molecular surface at the site of the crystallographic contacts discussed illustrated in panel D. This difference in structure may explain why DID-DD-CC constructs produce dimers while DID-DD constructs produce dimer-tetramer mixtures. (3.88 MB TIF) [file pone.0012896.s004.tif]

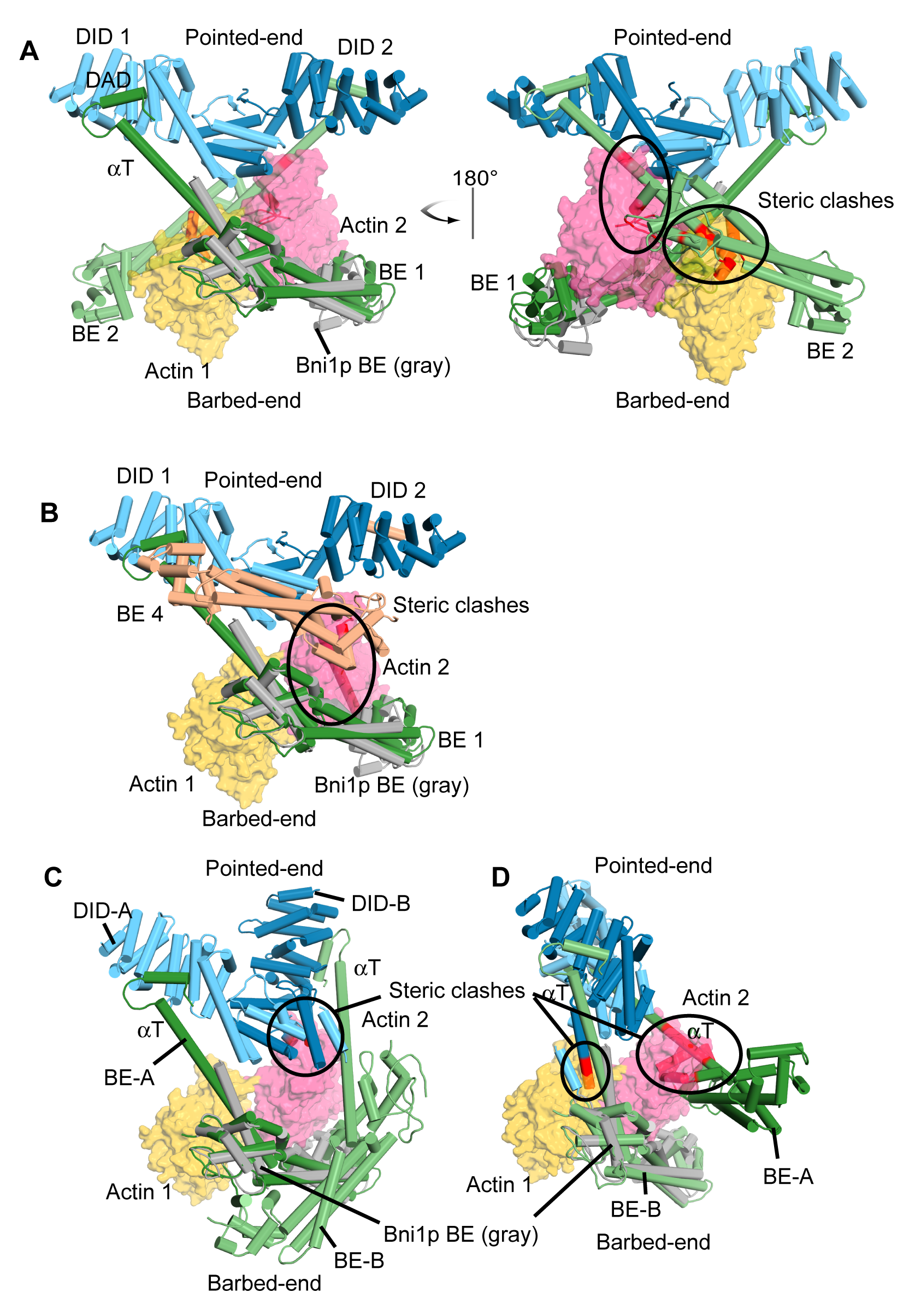

Supplement: Figure S5 — Demonstration of compatibility of actin binding to Models 1 and 4. Actin was modeled onto (A) BE1 of Model 1, (B) BE1 of Model 3, (C) BE-B of Model 4, and (D) BE-A of Model 4 (C) by superimposing the structure of the Bni1p bridge element bound to two actin molecules (PDB code; 1Y64). Actin 1 in yellow is bound to the post site of the Bni1p BE and Actin 2 in pink to the knob site. Actin 1 and 2 are related by pseudo two fold symmetry [21]). Observed steric clashes with actin are indicated by red colored parts of mDia1. DAD is not colored in red only in this figure for clarity. In (A), major clashes occur between both actin molecules and the other bridge (BE2) but not with DID-DD. In (B), Actin 2 clashes with BE4. In (C), Actin 2 but not Actin 1 clashes with DD. In (D), Actin 2 clashes with BE-A but not with DID-DD and CC potentially clashes with Actin 1. (2.84 MB TIF) [file pone.0012896.s005.tif]

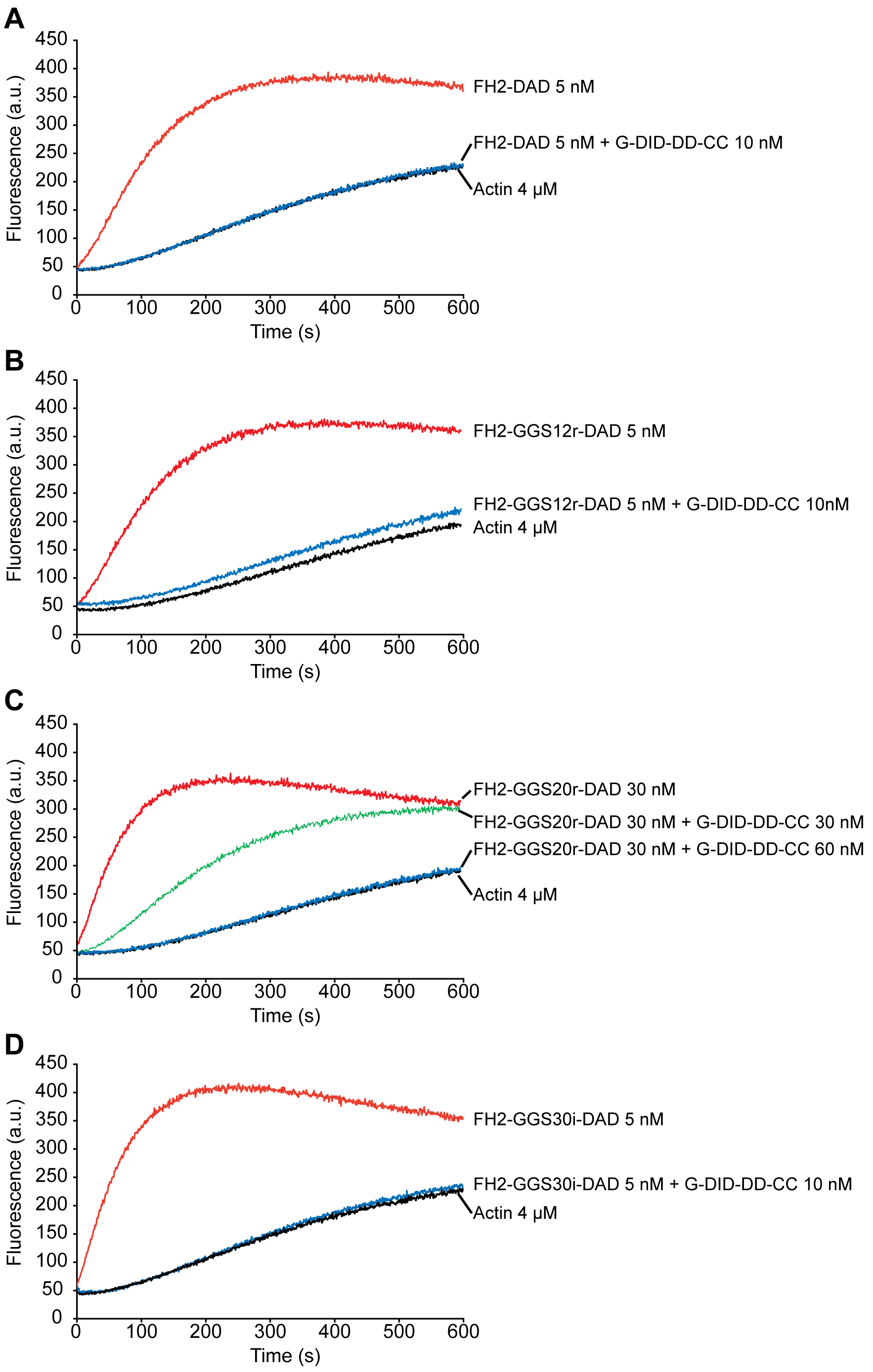

Supplement: Figure S6 — Introducing flexibility in αT does not affect inhibition by the N-terminus. Inhibition of (A) FH2-DAD wild type or (B-D) artificial constructs by the G-DID-DD-CC dimer was tested in actin assembly assays. In (B), FH2-GGS12r-DAD, in which 12 residues in αT (1160–1171) were replaced with 4 repeats of Gly-Gly-Ser sequence, shows actin assembly activity at a similar level as wild type and can be inhibited. In (C), FH2-GGS20r-DAD including replacement of 20 residues (1152–1171) shows slightly less activity and can also be inhibited. In (D), FH2-GGS30i-DAD, in which 10 repeats of Gly-Gly-Ser sequence was inserted between 1159-1160, shows similar activity and can also be inhibited. (0.66 MB TIF) [file pone.0012896.s006.tif]

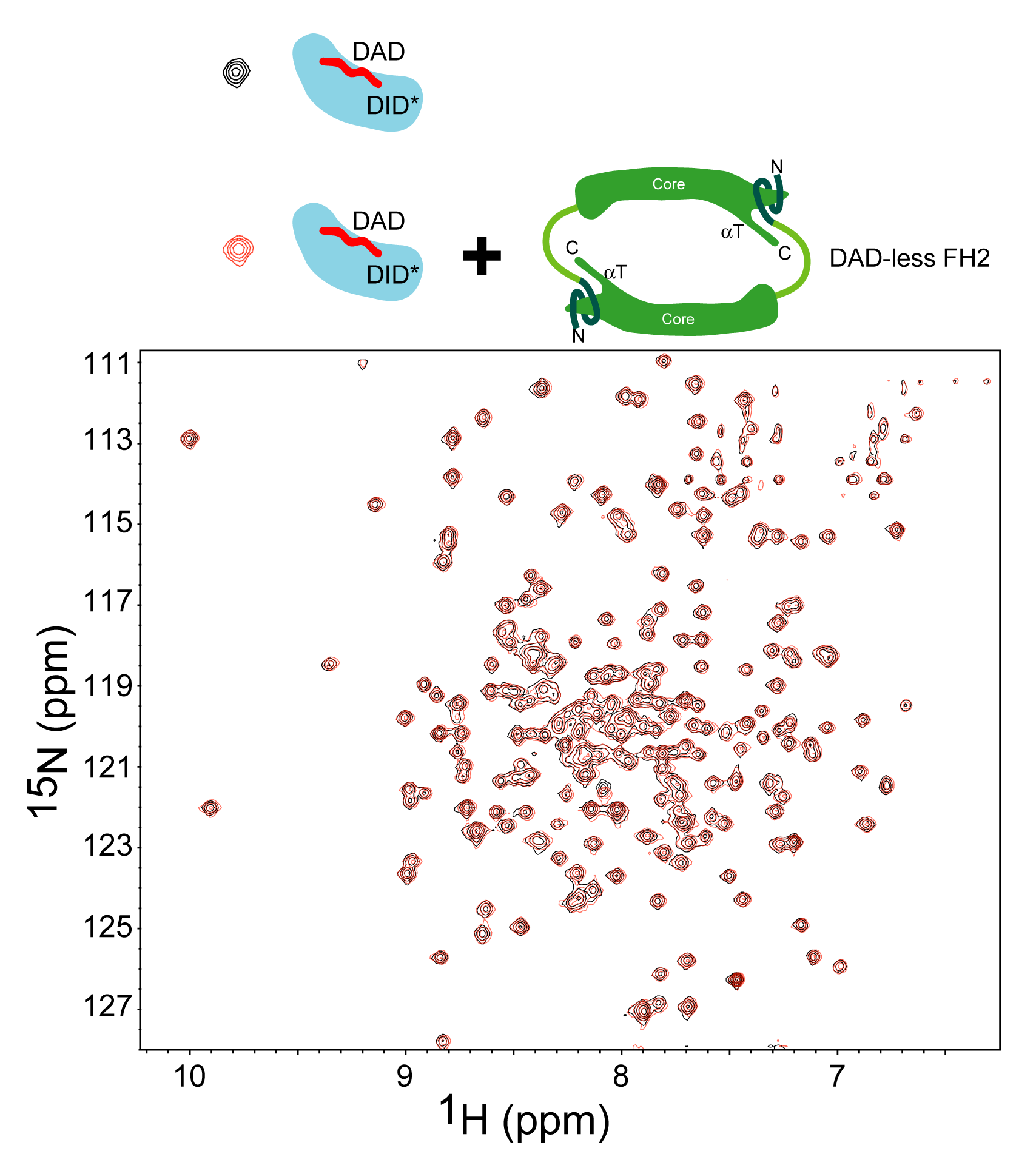

Supplement: Figure S7 — Isolated DID•DAD complex does not interact with the FH2 domain. An overlay of the 1H/15N HSQC spectra of the complex of 15N-DID bound to unlabeled DAD shows no significant changes in peak position or linewidth upon addition of unlabeled FH2 domain (lacking DAD). Because of the high molecular weight of the FH2 domain, even weak interactions should have caused observable peak broadening. Thus it is very likely that the FH2 domain does not interact with DID at a measurable affinity when it is not tethered to the N-terminus through the linked DAD. (0.72 MB TIF) [file pone.0012896.s007.tif]

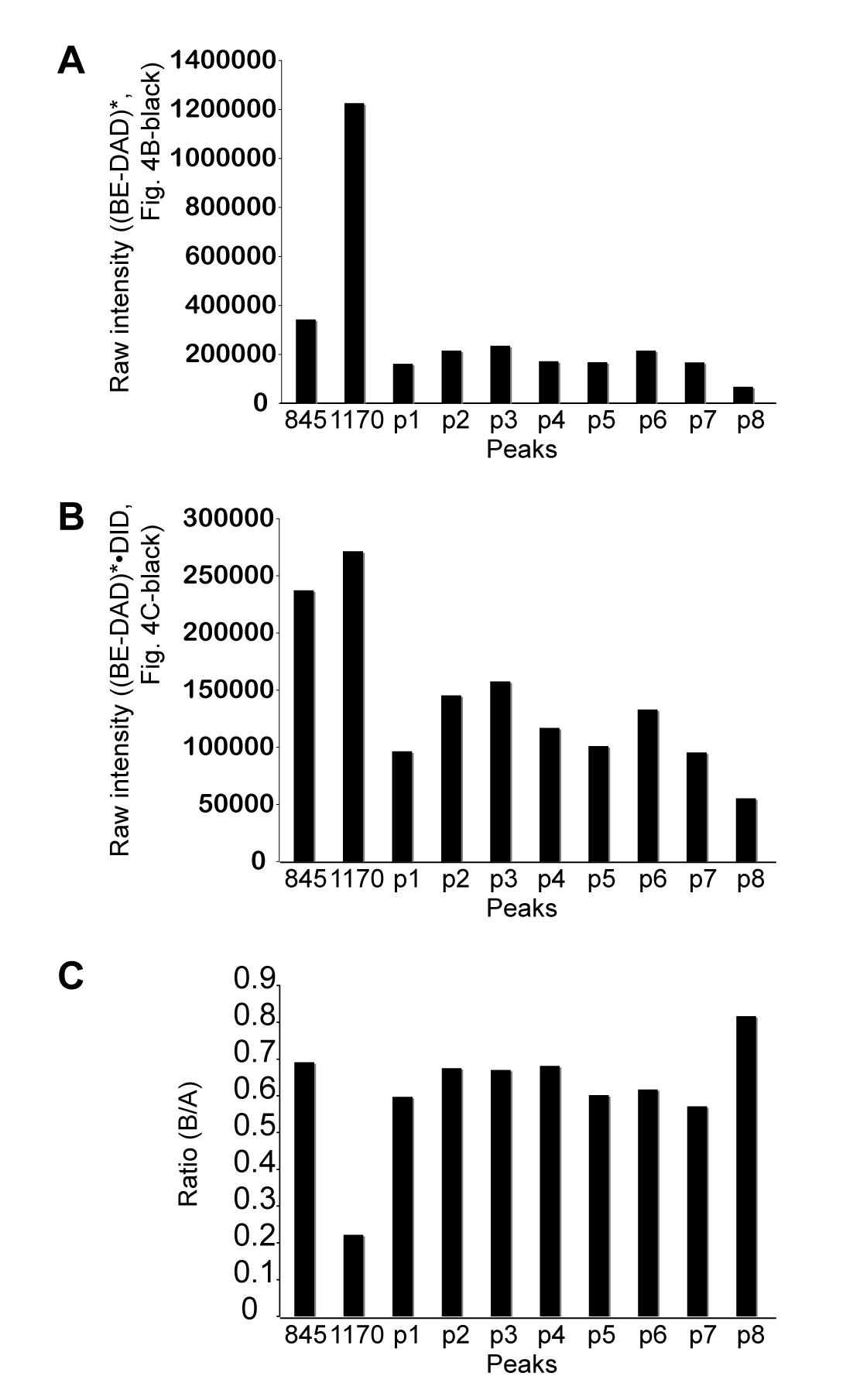

Supplement: Figure S8 — Peak intensities in the 1H-13C Ile δ1 methyl-TROSY HMQC spectra of BE-DAD in Figure 4. Raw peak intensities of the spectra of BE-DAD in the (A) absence or (B) presence of unlabeled DID (both in the absence of TMR-actin and shown in black in Figs. 4B and 4C). In (C), the ratios of the intensities in (B) divided by those in (A) for each peak are shown. The higher intensity of the Ile1170 resonance in (A) indicates that the DAD region is highly mobile in the free BE-DAD protein. In the absence of actin, the Ile845 resonance is affected by DID to the same extent as the unassigned peaks 1-8 (which represent residues in the core of the BE), while the Ile1170 resonance is greatly decreased on intensity (panel C). This is consistent with the DID differentially decreasing the mobility of Ile1170 in the DAD region. This behavior contrasts with that shown in Figures 4B and C, where actin differentially broadens the Ile845 resonance. (0.28 MB TIF) [file pone.0012896.s008.tif]
